# The Impact Evaluation of Acid Mine Drainage on Zebrafish (*Danio rerio*) and Water Fleas (*Daphnia magna*) in the Vicinity of the Geum River Basin in Korea

**DOI:** 10.3390/ijerph192416470

**Published:** 2022-12-08

**Authors:** Hyojik Yoon, Jonghyun Yoon

**Affiliations:** 1 Institute of Natural and Science, College of Science and Technology, Korea University, Sejong 30019, Republic of Korea; 2 National Institute of Environmental Research, Incheon 22689, Republic of Korea

**Keywords:** acid mine drainage, bioassay, cognitive function, locomotion, zebrafish

## Abstract

Heavy metals, such as copper, lead, and cadmium, carried by acid mine drainage are pollutants of the aquatic ecosystem, posing a significant health risk to the water resource for humans. Environmental technologies to reduce metal contamination are applied for post-mining prevention and improvement. Despite detailed pollution management, water contaminated by heavy metals still flows into the natural water system. This study investigated the impact of drainage discharged from abandoned mines near the major river in South Korea on aquatic organisms. The toxicity of the field water showed a more significant effect than observed through the experiment for each heavy-metal concentration. Various toxic substances coexisted in the field water around the mine, such that the overall toxic intensity was high even when the concentration of each heavy metal was low. As a result, the inhibition of activity of aquatic organisms was observed at low individual concentrations, and further investigation on the effect of long-term exposure to trace amounts of heavy metals is required.

## 1. Introduction

Metal mining produces heavy metals in aquatic ecosystems [1]. After mining, metal leak prevention management should be performed as part of environmental protection [2] to protect surface water and groundwater systems. Despite this effort, acid mine drainage (AMD) can flow into rivers, adversely affecting the entire aquatic ecosystem and, ultimately, humans through biological accumulation [3,4]. Pollution from abandoned mines is determined by various physical, chemical, and geographic factors specific to each site, as well as local meteorological (rainfall) factors. The heavy metals in mine drainage are deposited as sediments or absorbed in the receiving river sediments and are concentrated at extremely high levels [5]. Several studies have observed that as rainfall intensity increases, heavy-metal concentrations naturally increase in the receiving bodies of water [6,7,8]. Heavy metals carried by AMD are potentially lethal to humans and other organisms, as they are dispersed in the environment through transport media, such as water, soil, and the atmosphere [9,10,11]. Therefore, the removal and management of AMD sources are necessary to develop countermeasures against the contamination of leachate-containing heavy metals [12]. However, effective post-management programs for these abandoned mines require a clear understanding of the behavior of pollutants based on various factors and approaches in terms of pollutant loads.

Moreover, the current ecological assessment methods are not sufficiently sensitive, particularly for trace concentrations of pollutants. Previous research provided a procedure to estimate the overall health of the local water system by analyzing the conditions and diversity of the collected species [12]. Recently, metabolomics has become an essential tool for understanding the mechanisms of toxicity because it can provide valuable insights into changes in the health of living organisms upon exposure to stimuli with greater sensitivity [13,14]. This study aimed to quantify the heavy-metal pollution load in the bodies of water near three abandoned mines and to assess the toxicity of these heavy metals through exposure tests using zebrafish (*Danio rerio*) and planktonic crustacean (*Daphnia magna*). Zebrafish (*Danio rerio*) are easy to handle embryologically and genetically due to the rapid growth characteristics of the embryo. In addition, they offer economic advantages over mammalian species that are primarily used as genetic models in medicine, biotechnology, and pharmacology. Therefore, zebrafish are used as a vertebrate animal model in various research fields; in particular, they are used for anatomical, physiological, genetic, developmental, and behavioral studies in visual neuroscience [15]. Because the zebrafish egg is transparent and some zebrafish have been genetically manipulated to have a fluorescent organ, they are easily accessible during all stages of development, from embryo to adult [16].

Toxicity was quantified based on the effect of exposure to AMD on the behavioral and cognitive abilities of zebrafish larvae.

## 2. Materials and Methods

### 2.1. Site Description and Water Sampling

**Figure 1:**
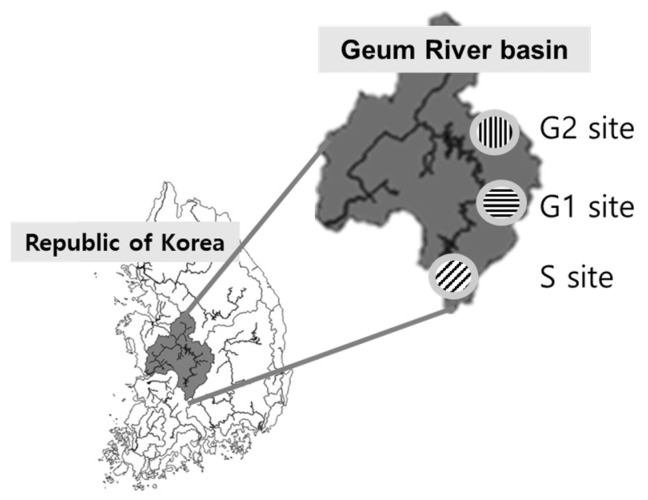
Location of the research area.

The Geum River, one of the major rivers in the Republic of Korea, was chosen as the study area. Mines in the Geum River Basin that could affect the water source conservation area (Figure 1 and Table 1) were classified. The G1 site was a gold and silver mining area. The S-site mine contained galena, sphalerite, quartz, and calcite. The G2 mine primarily mined copper, zinc, and lead. Eight water samplings were carried out, five times during the dry period and three times during rainfall. Based on location data, the flow rate and pollutants flowing into the nearby river from the three surveyed mines were investigated. The runoff distances from each mining area to the nearest river were 495 m in G1, 1460 m in S, and 380 m in G2, respectively. The water was sampled from the middle of the stream. In each sampling, the grab-sampling method was used. The water samples were collected in the period from March 2016 to September 2016. The water quality indicators (DO, pH, EC, TDS, and ORP) were measured and recorded onsite. The water (1000 mL) was filled in a sterilized sample bottle and spiked with 2 mL nitric acid and sealed after removing the air. The concentrations of the metals were determined using inductively coupled plasma mass spectrometry (ICP-MS, II Perkin ELAN-DRC, Waltham, MA, USA).

### 2.2. Zebrafish

Zebrafish (*Danio rerio*) are used in toxicity evaluation because they have a preferred wavelength. Zebrafish hatch within 2 to 3 days after spawning, and 5 days after birth, the visual organ is developed such that the wavelength of color can be distinguished.

Zebrafish that have begun to distinguish colors have a characteristic of preferring a short-wavelength region (blue) rather than a long-wavelength region (yellow), which has been proven experimentally by previous work [17].

### 2.3. Cognitive Function and Locomotion Change Test

The experimental concentrations were selected based on a river water quality survey. The highest concentration of each metal detected was set as the exposure concentration (Copper: 1000 μg/L; Pb: 100 μg/L; Cd: 1000 μg/L; As: 50 μg/L; Zn: 100,000 μg/L).

The tests were performed with copper, lead, cadmium, arsenic, and zinc, respectively.

Zebrafish can detect visible light domains similar to those of humans. Zebrafish have specific preferences and wavelengths. Using these zebrafish attributes, researchers measure and quantify behavioral changes by analyzing the number of zebrafish larvae that remain at the location of the preferred wavelength for half an hour.

A color maze system was installed to monitor the zebrafish to determine the manner in which behavioral changes are affected by exposure to small amounts of heavy metals. 

**Figure 2:**
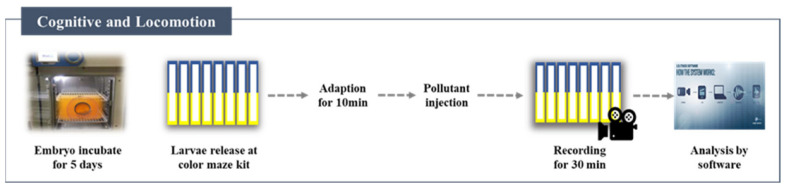
Zebrafish cognitive and locomotive change test model.

Zebrafish preferred when the visible light zone was set to blue wavelengths and the secret visible light zone was set to yellow wavelengths (Figure 2). Ten zebrafish larvae were placed in each color maze kit (Genomic Design^TM^, Daejeon, Republic of Korea); the temperature and light intensity were adjusted. Since the larvae for each heavy-metal experiment were obtained from a new embryo tank, a control experiment was required for each tank. As a control group, a clean culture solution was applied. The movement of larvae in a clean culture solution without pollutants was set as a control. A digital camcorder was used for 30 min to record the larvae after they were exposed to heavy metals. The Lolitrack 4.1 (Loligo^®^ systems, Viborg, Denmark) was used to analyze locomotive changes. Additionally, the average velocity, acceleration, and active time were analyzed. The experiment was repeated three times with new larvae.

### 2.4. Daphnia magna Bioassay Test

To evaluate the toxicity of *Daphnia magna*, breeding conditions were established according to the standard toxicity test method [18]. The culture medium was aerated overnight after injecting KCl, MgSO_4_, and NaHCO_3_ into 18 L DI water. Subsequently, CaSO_4_·2H_2_O was added to 2 L of distilled water and dissolved. The two solutions were mixed and used as the culture medium. The culture conditions were a water temperature of 20 ± 2 °C, a light/dark cycle of 16 h:8 h, and a light intensity of 500–1000 lux. The culture medium was changed three times per week.

The population of *D. magna* increased after continuous subculture. Therefore, in this study, we performed a bioassay by setting the monitoring items for *D. magna*. The bioassay used as a risk assessment in the environment analyzes the effects of biological function reduction, swimming inhibition, and lethality of the target species when exposed to a specific environmental substance.

### 2.5. Statistical Analysis

All statistical analyses were conducted using SPSS (IBM SPSS 21) software (Chicago, IL, USA). A probability of *p* < 0.05 was accepted as statistically significant using a *t*-test. ANOVA analysis was applied to analyze the impact of differences between monitoring sites.

## 3. Results and Discussion

### 3.1. Analysis of Stream Water

The rivers surrounding the G1 site are contaminated with cadmium and zinc (Table 2). The pollution concentration primarily increases after rainfall (August and September), probably owing to mine runoff. Severe cadmium and zinc contamination was also discovered in river water near the G1 area. However, copper, arsenic, and lead did not exceed the water quality standards. Cadmium and zinc exceeded the standard for river water quality by 7–14% and 7%, respectively. Rivers near G2 were contaminated with cadmium, copper, and zinc, even during the non-rainy season. Copper and zinc exceeded the standard for water quality. In rivers near the S site, cadmium exceeded the water quality standard by 64–78%. The standards were consistently exceeded for lead and zinc, similar to cadmium. Copper, arsenic, mercury, and nickel concentrations either did not exceed water quality standards or were not detected. In the S region, the pollution range was widely distributed compared with the G1 and G2 sites. This is believed to be due to the topographical characteristics of the river flowing along a steep continuation.

### 3.2. Behavioral Changes of Zebrafish

#### 3.2.1. Inhibition of Zebrafish Preferred-Wavelength Discrimination by Exposure to Heavy Metals

**Figure 3:**
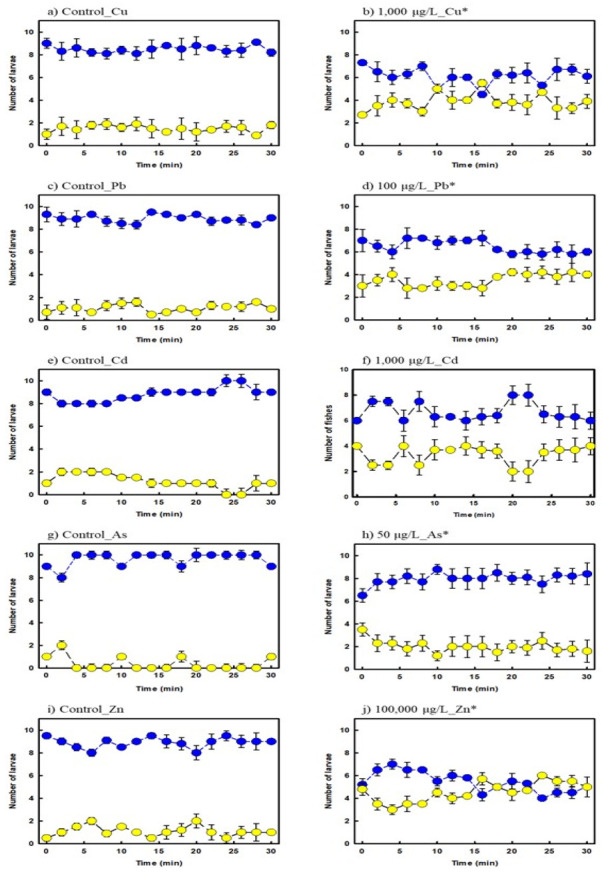
Cognitive function changes of zebrafish larvae after heavy-metal exposure (blue; preferred region, yellow; non-preferred region, * *p* < 0.05).

The zebrafish larvae exposed to copper tended to remain in the non-preferred wavelength region (yellow circle) compared to the control at low concentrations (Figure 3a,b). However, when the wavelength position was switched after 30 min, the cognitive ability normalization rate was higher than that of the relatively high-concentration exposure group; therefore, the position ratio in the preferred wavelength region (blue circle) increased. Exposure to 1000 μg/L of copper caused cognitive impairment in the inability to distinguish preferred wavelengths (blue) over time. Lead exposure changed (Figure 3c,d) swimming behavior.

A relatively unstable swimming time, at the preferred wavelength, was observed at 10 μg/L and 100 μg/L compared to the control group. As the concentration increased to 100 μg/L, the swimming activity reduced, and fish trying to escape from the non-preferred wavelength region were not detected. After 30 min of observation, cognitive impairment was consistently confirmed at concentrations of 100 μg/L or higher, and the recovery of cognitive ability did not occur. Figure 3e,f present the experimental results for cadmium.

As the cadmium concentration increased, the positional trend standard deviation of zebrafish larvae changed significantly. It was observed that increased cadmium exposure over time resulted in an increased fluctuation range for the preferred wavelength of zebrafish, which affects the cognitive ability of larvae. Arsenic is a toxic heavy metal; it severely harms various organs, as well as the central nervous system. Significant side effects that inhibit neurotransmission in the brain occur; therefore, arsenic concentrations should be monitored continuously. Compared to other heavy metals, the standard deviation of the preferred wavelength (blue circle) was relatively large, even below 50 μg/L (Figure 3g,h). No changes in behavior were observed. It is believed that the tendency to move from the initial position did not occur as, based on arsenic exposure, swimming sharply decreased.

Zinc is a relatively weak metal. However, excessive zinc influx into nerve cells leads to nerve damage. The zinc concentration in the water sample was more than 100 times higher than that of other metals, which may have an effect on the aquatic ecosystem. Below 1000 μg/L of zinc, no significant cognitive impairment was observed (Figure 3i,j). When exposed to 5000 μg/L or more, cognitive impairment increased by approximately 20%, and at 100,000 μg/L, the incidence of cognitive impairment increased by 50% or more compared to the control group.

#### 3.2.2. Locomotion Changes by Exposure to Heavy Metal

**Figure 4:**
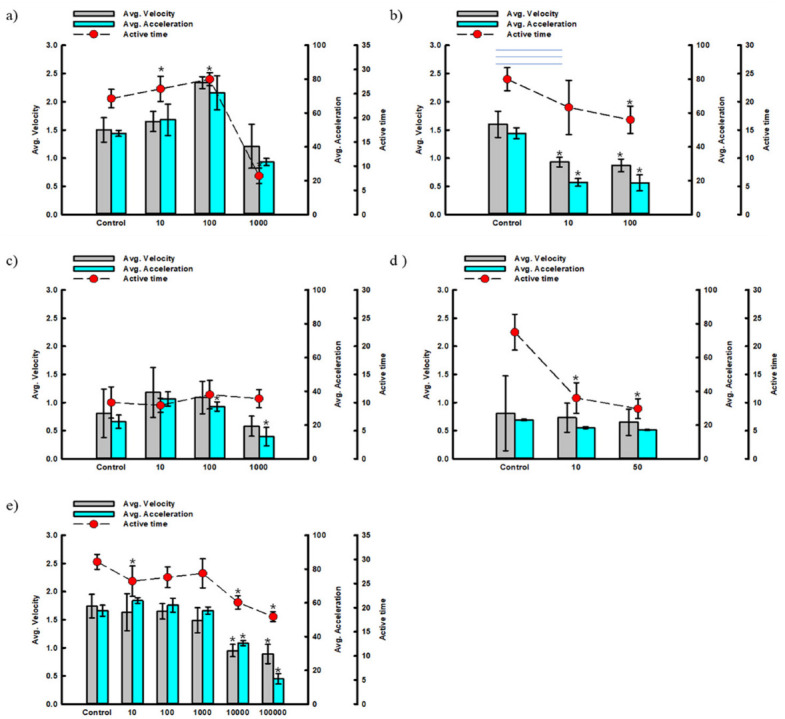
Locomotive changes of zebrafish larvae by heavy-metal exposure (* *p* < 0.05, (**a**) Cu, (**b**) Pb, (**c**) Cd, (**d**) As, (**e**) Zn).

Organisms escape from predators in aquatic ecosystems by changing the velocity, acceleration, and activity time [19]. To analyze the locomotion of the larvae, the average velocity, average acceleration, and active time were measured and compared. The analysis results for each heavy metal substance are as follows. First, copper changed locomotion at concentrations of 10 and 100 μg/L. Compared to the control group, the average velocity increased by 10–56%, acceleration by 16.7–50%, and activity time by 8.3–16.7%. The recovery time after exposure was prolonged, and the overall locomotion was not significantly affected. However, when exposed to 1000 μg/L, it decreased by 19.3, 35.4, and 66.7% for all observation factors, respectively (Figure 4a).

Lead showed a decreasing trend for all items, despite the low exposure concentrations. Compared to the control group, the speed decreased by 41.9–45.6%, acceleration by 60.4–61.0%, and activity time by 20.8–30%, respectively. Lead toxicity reduces brain activity [20]. It was confirmed that it played a significant role in reducing the brain function that controls swimming and changes in cognitive ability (Figure 4b).

In general, cadmium toxicity causes cancer and respiratory, liver, and kidney diseases or interferes with calcium intake. No appreciable change was observed after exposure to 10–100 μg/L concentrations. However, when exposed to concentrations greater than 1000 μg/L, the velocity and acceleration decreased by 28.4% and 40.5%, respectively. It was indirectly confirmed that the effect on the body gradually increased as the concentration or time of exposure to cadmium increased. As mentioned in Section 3.2.1, arsenic showed little change in all areas, with the active time reduced by up to 60.4% (Figure 4c). 

The arsenic exposure test showed a relatively low value compared to that of other heavy metals. Thus, the effect of arsenic on the aquatic ecosystem can be stronger than that of other heavy metals (Figure 4d). Compared with other substances, zinc showed toxic effects at high concentrations. When exposed to concentrations greater than 10,000 μg/L, the velocity and acceleration were reduced by more than 30%. Although high zinc concentrations affected cognitive ability, they had a relatively low effect on motility changes (Figure 4e).

### 3.3. Field Water Exposure Test

#### 3.3.1. Inhibition of Zebrafish Preferred-Wavelength Discrimination by Exposure to Real Water

**Figure 5:**
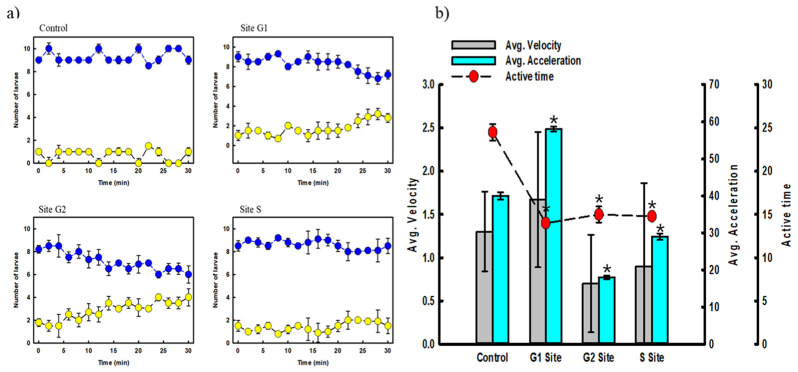
(**a**) Cognitive function, and (**b**) locomotion changes of zebrafish larvae (Real-site sample) (blue; preferred region, yellow; non-preferred region, * *p* < 0.05).

Lab-scale exposure experiments (Section 3.2) may show different results from actual field experiments. Therefore, the same experimental method was used to examine the effect of river water around the mine, wherein various types of heavy metals are mixed, on zebrafish larvae. For field water, the sample with the highest degree of contamination was selected and used for the experiment. From lab-scale experiments, it was expected that the toxicity evaluation of the field water itself would be complex, as it affected the behavior of zebrafish even when the concentration of heavy metals was 10 μg/L or lower. At the G1 site, there was no significant difference from the control group when exposed to contaminated field water for the first 30 min. However, the discrimination ability for the preferred wavelength decreased as the exposure time increased, and a change in the ability, up to 20%, was observed. In the G2 region, the discrimination ability decreased by 15% with increasing exposure time. However, owing to the strong neurotoxicity of arsenic, the time taken to develop cognitive impairment was reduced by 20%. In the S region, the zinc concentration was approximately 10 times higher than that in the other regions. Nevertheless, cognitive impairment was not observed, as zinc has relatively weak toxicity compared to other heavy metals (Figure 5a).

#### 3.3.2. Locomotive Changes by Exposure to Water

At the G1 site, with water exposure, activity time decreased by 42.9%, although the average speed and acceleration increased by 28.5% and 45%, respectively, compared with the control group (Figure 5b). The inhibition of activity showed the most significant effect at the G2 and S sites. A decrease occurred in all items compared with the control group, and a reduction in the acceleration of up to 55% was observed. In the G2 region, more arsenic was detected compared to other mine samples. In the S region, the zinc concentration was 10 times higher than that of the other samples. Among the motion analysis parameters, the activity time item showed the same trend as the results of the cognitive observation experiment. It is believed that a correlation exists between the change in cognitive intelligence and the activity-time relationship of fish; therefore, precise verification is required through further research. As a result of ANOVA analysis, the significance probability for the other items, except average velocity, was lower than 0.05. The contaminated field river water was confirmed to have an impact on zebrafish locomotion.

### 3.4. Daphnia magna Bioassay Test

To conduct a general toxicity test on field water, toxicity sensitivity was assessed using *D. magna* (Table 3). Using the EC50 as the criterion, the same TU results were obtained, and the highest ecotoxicity was confirmed in the G2 and S sites as a result of the TU calculation. In the case of the S site, all *D. magna* swimming inhibition occurred at 100% raw water concentration. As a result, the toxicity unit was 3.00. At the G2 site, similar to the S region, a 3.10 value was obtained. The study results for zebrafish and the swimming inhibition rate of *D. magna* showed similar trends.

## 4. Conclusions

Abandoned mines, which are widely distributed around the world, cause environmental pollution due to various factors. Mine drainage pollution could be caused by rainfall, water flow, or mountain slope. Drainage would be introduced into the nearby natural water system. Pollutants in mine drainage pose serious threats to aquatic ecosystems. Contaminants are mostly composed of heavy metals, so the harmful effects on the water environment must be clearly analyzed.

The leakage of heavy metals shows immediate toxicity to the ecosystem; however, it is believed that the environment, over time, can adapt to this toxicity level [21]. Despite adapting, its impact on the ecosystem is an area that requires continuous observation.

In this study, it was confirmed that AMD toxicity that can occur in mines was similarly expressed in zebrafish (*Danio rerio*) and water fleas (*D. magna*), the species to be evaluated for toxicity.

However, although it was detected in Danio rerio, it was confirmed that the TU value for *D. magna* was not detected. This means that the existing ecotoxicity assessment may overlook the toxicity of trace heavy metals in the water system.

Through these results, it is possible to suggest that the environmental impact can be analyzed more accurately if a comprehensive toxicity evaluation of the two species is performed on field water samples containing complex contaminants.

Nevertheless, since this research method also has uncertainty due to statistical values, it is necessary to continuously develop advanced toxicity evaluation methods in order to quantify harmful toxicity.

## Figures and Tables

**Table 1:** The latitude and longitude of research areas (Sampling point).

Mining Site	Latitude	Longitude
G1	35°54′22.7″ N	127°44′45.4″ E
G2	36°20′08.6″ N	127°43′52.9″ E
S	35°57′00.3″ N	127°40′15.1″ E

**Table 2:** Analysis of contaminated water samples in each monitoring site.

		Concentration (N.D: Not Detected)										
		Cd (μg/L)	Cu (μg/L)	As (μg/L)	Hg (μg/L)	Pb (μg/L)	Ni (μg/L)	Zn (μg/L)	pH	EC (μS/cm)	DO (mg/L)	TDS (mg/L)
River water quality standards in Korea		5		50		50			6.5–8.5		7.5	
Dry Period	G1	6.42 ± 1.17	40.31 ± 28.56	12.82 ± 3.62	N.D	6.85 ± 2.05	N.D	711.00 ± 58.00	6.07 ± 0.79	302.14 ± 90.33	8.81 ± 0.93	207.99 ± 59.31
	G2	1253.6 ± 67.84	38,942 ± 5229.82	N.D	N.D	83 ± 25.25	N.D	127,994 ± 3533.29	7.31 ± 0.39	181.28 ± 3.94	6.29 ± 1.26	117.91 ± 2.52
	S	31.40 ± 6.50	10.8 ± 3.63	N.D	N.D	61.40 ± 40.78	N.D	4732 ± 1173.82	7.84 ± 0.08	227.2 ± 52.49	7.36 ± 1.14	115.27 ± 20.54
Rainfall period	G1	4.38 ± 0.31	74.20 ± 19.93	7.80 ± 3.97	N.D	39.40 ± 23.31	N.D	488.80 ± 52.38	7.26 ± 0.08	365.28 ± 21.98	9.39 ± 0.23	279.23 ± 7.69
	G2	6.94 ± 1.49	16.96 ± 8.51	14.82 ± 4.21	N.D	3.04 ± 0.91	N.D	614.70 ± 59.45	7.32 ± 0.41	180.18 ± 3.49	5.03 ± 0.90	149.60 ± 39.15
	S	33.20 ± 7.73	7.80 ± 4.62	N.D	N.D	136.00 ± 64.33	N.D	5402.20 ± 785.56	7.52 ± 0.41	242 ± 59.17	7.44 ± 0.89	174.48 ± 42.66

**Table 3:** The bioassay test using *D. magna* by field water exposure.

Mining Site	Exposure Concentration	Immobilization Reduction	EC50 (%)	TU
G1	6.25%	1.33 ± 0.58	55.12	1.81
	12.5%	2.33 ± 0.58		
	25%	4.00 ± 1.00		
	50%	7.33 ± 0.58		
	100%	11.00 ± 1.00		
G2	6.25%	4.00 ± 1.00	32.3	3.10
	12.5%	7.00 ± 0.00		
	25%	10.67 ± 1.15		
	50%	16.67 ± 0.58		
	100%	19.33 ± 0.58		
S	6.25%	3.33 ± 0.58	33.32	3.00
	12.5%	7.67 ± 0.58		
	25%	11.00 ± 1.00		
	50%	17.00 ± 1.00		
	100%	19.67 ± 0.58		

## Data Availability

Not applicable.
